# Are China’s oldest-old living longer with less disability? A longitudinal modeling analysis of birth cohorts born 10 years apart

**DOI:** 10.1186/s12916-019-1259-z

**Published:** 2019-02-01

**Authors:** Zuyun Liu, Ling Han, Qiushi Feng, Matthew E. Dupre, Danan Gu, Heather G. Allore, Thomas M. Gill, Collin F. Payne

**Affiliations:** 1 Department of Pathology, Yale School of Medicine, New Haven, CT USA; 2 Department of Internal Medicine, Yale School of Medicine, New Haven, CT USA; 3 Department of Sociology, National University of Singapore, Singapore, Singapore; 4 Department of Population Health Sciences, Duke University, Durham, NC USA; 5 Department of Sociology, Duke University, Durham, NC USA; 6 Independent Researcher, New York, NY USA; 7 School of Demography, Research School of Social Sciences, Australian National University, 9 Fellows Road, Acton, ACT, Canberra, 2601 Australia

**Keywords:** Aging, Disability, Mortality, Birth cohort, Oldest-old, Life expectancy, China

## Abstract

**Background:**

China has transitioned from being one of the fastest-growing populations to among the most rapidly aging countries worldwide. In particular, the population of oldest-old individuals, those aged 80+, is projected to quadruple by 2050. The oldest-old represent a uniquely important group—they have high demand for personal assistance and the highest healthcare costs of any age group. Understanding trends in disability and longevity among the oldest-old—that is, whether successive generations are living longer and with less disability—is of great importance for policy and planning purposes.

**Methods:**

We utilized data from successive birth cohorts (*n* = 20,520) of the Chinese oldest-old born 10 years apart (the *earlier* cohort was interviewed in 1998 and the *later* cohort in 2008). Disability was defined as needing personal assistance in performing one or more of five essential activities (bathing, transferring, dressing, eating, and toileting) or being incontinent. Participants were followed for age-specific disability transitions and mortality (in 2000 and 2002 for the *earlier* cohort and 2011 and 2014 for the *later* cohort), which were then used to generate microsimulation-based multistate life tables to estimate partial life expectancy (LE) and disability-free LE (DFLE), stratified by sex and age groups (octogenarians, nonagenarians, and centenarians). We additionally explored sociodemographic heterogeneity in LE and DFLE by urban/rural residence and educational attainment.

**Results:**

More recently born Chinese octogenarians (born 1919–1928) had a longer partial LE between ages 80 and 89 than octogenarians born 1909–1918, and octogenarian women experienced an increase in partial DFLE of 0.32 years (*P* = 0.004) across the two birth cohorts. Although no increases in partial LE were observed among nonagenarians or centenarians, partial DFLE increased across birth cohorts, with a gain of 0.41 years (*P* < 0.001) among nonagenarians and 0.07 years (*P* = 0.050) among centenarians. Subgroup analyses revealed that gains in partial LE and DFLE primarily occurred among the urban resident population.

**Conclusions:**

Successive generations of China’s oldest-old are living with less disability as a whole, and LE is expanding among octogenarians. However, we found a widening urban-rural disparity in longevity and disability, highlighting the need to improve policies to alleviate health inequality throughout the population.

**Electronic supplementary material:**

The online version of this article (10.1186/s12916-019-1259-z) contains supplementary material, which is available to authorized users.

## Background

More than 23 million oldest-old individuals (those aged 80+) live in China, contributing 18% of the oldest-old population worldwide in 2015. As the Chinese population ages over the coming decades, this percentage is expected to rapidly grow, and by 2050 over a quarter of the global oldest-old population will live in China (based on the UN’s medium variant projection) [1]. The oldest-old are a highly vulnerable population in China and are among the most policy-neglected and socially disadvantaged people [2]. They are very likely to require assistance in their essential activities of daily living (ADLs), posing challenges for their families and caregivers [3, 4]. The complex healthcare needs of the oldest-old (including disability, chronic diseases/multimorbidity, and cognitive impairment) are a substantial demand on the health system of China [5–7]. Consequently, this age group has the highest rates of healthcare expenditure and has also seen the fastest rise in healthcare expenditure in recent decades [8].

There is a substantial concern from policymakers worldwide on how to manage and care for this expanding vulnerable population, and a need for more detailed insights into how the care needs of these oldest-old populations are changing. This can be, at least in part, informed by evaluating whether they are living longer and with less disability across successive generations. Two major views on the trends of morbidity among older adults have been proposed. The first is the compression of morbidity hypothesis [9, 10], which states that the age of onset of morbidity or disability can rise more rapidly than life expectancy (LE) [11], thus resulting in a shorter period of morbidity prior to death. A contrasting theory, the expansion of disability hypothesis, posits that reductions in mortality (and thus, increases in LE) may result in a shift in frailty over successive cohorts, as individuals with worse health are now likely to survive longer than they would have in the past [12, 13], resulting in populations of older individuals with higher rates of disability in older ages [12–15]. Importantly, both of these theoretical viewpoints are focused primarily on understanding trends in morbidity and mortality among older adults ages 65–80, where the bulk of each occurs in most populations [11]. The ways in which these theoretical frameworks function in the oldest-old populations are unclear, as these high-longevity individuals have already far outlived their cohort’s LEs. In addition, these two viewpoints are not necessarily opposing—different sub-populations may experience expansions or compression of morbidity at the same time [16, 17], and different facets of health (disability, chronic diseases/multimorbidity, cognitive ability) may change in different directions across cohorts [16–18].

In China, several studies have evaluated trends in disability/morbidity over time [16, 19–23]. However, only one study by Zeng et al. [16] has focused on comparing how morbidity is changing across successive birth cohorts. Zeng and his colleagues compared three paired groups of Chinese oldest-old born 10 years apart (those 80–89, 90–99, and 100–105) at the time of survey in 1998 or 2008. They suggested that successive cohorts of oldest-old individuals are living longer, but found mixed evidence on whether or not a compression of disability and morbidity was occurring [16]. Though these findings provide useful information on cross-sectional changes in disability and morbidity of Chinese oldest-old over a 10-year period, their analyses did not account for the fact that disability is a dynamic condition in the later life [24]. Individuals relapse and remit between disabled and disability-free life, and the interactions between disability and mortality change over time and by individual characteristics [25]. Research investigating the compression/expansion of disability in the oldest-old needs to incorporate a dynamic view of cohort variations in disability. Accurately accounting for these transitions leads to a more nuanced understanding of the lived experience with disability and avoids generating biased estimates of the level of disablement at the population level [25]. In addition, though previous research has established that there are sociodemographic inequalities in disability and morbidity among older adults in China by urban/rural residence and socioeconomic status [23, 26–31], no research to date has explored how these inequalities have changed across birth cohorts. Given the rapid pace of economic and infrastructural development and the recent implementation of universal healthcare coverage in China, it is important to explore whether potential gains in LE (and potential reductions in disability) are being experienced evenly across the oldest-old population.

In this study, we utilized longitudinal survey data from a long-running study of 20,520 Chinese oldest-old and examined whether successive birth cohorts of octogenarians (aged 80–89 years), nonagenarians (aged 90–99 years), and centenarians (aged 100–105 years) are living longer and with less disability. We used a multistate life table (MSLT) model to estimate how partial LE (i.e., years lived between two ages, described in detail in the “Methods” section) and disability-free LE (DFLE) are changing within age groups over successive birth cohorts, a metric more directly applicable to understanding trends in population health than more commonly used measures focusing on changes over time periods. In addition, we explored whether there is sociodemographic heterogeneity in partial LE and DFLE by urban/rural residence and educational attainment.

## Methods

### Study population

Data for our analyses are from the Chinese Longitudinal Healthy Longevity Survey (CLHLS), one of the largest samples of oldest-old in the world. The details of the sampling design, response rates, attrition, and systematic assessments of data quality across numerous measures have been described elsewhere [32]. In brief, the CLHLS, initiated in 1998, is an ongoing nationwide survey of Chinese oldest-old individuals, with the aim of collecting a comparable sample of octogenarians, nonagenarians, and centenarians in a randomly selected half of counties/cities in 22 provinces throughout China [32]. The total population of the 22 provinces accounts for about 90% of the total population in China according to the 2010 census [33]. The CLHLS attempted to recruit all centenarians who agreed to participate in the sampled counties/cities. Using a targeted random-sampling design, the CLHLS interviewed approximately equal numbers of octogenarians and nonagenarians living near the centenarians (e.g., in the same villages or streets) [32]. This design supports the major aim of investigating determinants of healthy longevity of different age and sex groups who live in the same social and natural environment [16]. Extensive questionnaires were used to collect a comprehensive set of information, including sociodemographic characteristics, and ADLs. All information was obtained during in-home interviews that lasted about 2 h. Each participant provided a written informed consent. The informed consent was signed by the next-of-kin when the participant was not able to write.

The date of death was collected from official death certificates when available; otherwise, the next-of-kin and local residential committees were consulted. An analysis by Gu and Dupre [34] demonstrated that the single-age-sex-specific mortality rates at oldest-old ages including centenarians in CLHLS fit well with the Kannisto model, a function that has been shown to best fit mortality trajectories at oldest-old ages in various countries with high-quality data [35]. According to previous reports [36–38], the CLHLS has documented good data quality, including assessments of mortality, age reporting, proxy response, sample attrition, and reliability and validity of major health measures.

As described in a previous study [16], three paired birth cohorts (*earlier* vs. *later* cohorts) who were born 10 years apart were interviewed at the same age in 1998 and 2008. For paired age group 1, we compared participants born in 1909–1918 vs. 1919–1928 (octogenarians, aged 80–89 years in 1998 vs. 2008); for paired age group 2, we compared participants born in 1899–1908 vs. 1909–1918 (nonagenarians, aged 90–99 years in 1998 vs. 2008); and for paired age group 3, we compared participants born in 1893–1898 vs. 1903–1908 (centenarians, aged 100–105 years in 1998 vs 2008). For the *earlier* cohort (i.e., hereafter refer to as octogenarians, nonagenarians, and centenarians in 1998), follow-up information from the 2000 and 2002 waves were used to estimate the conditional probabilities of experiencing disability transitions and mortality. For the *later* cohort (i.e., octogenarians, nonagenarians, and centenarians in 2008), follow-up information from the 2011 and 2014 waves were used to obtain similar estimates.

### DFLE

Disability was assessed by the Katz index [39] that included six essential ADLs: bathing, transferring, dressing, eating, toileting, and continence. The Chinese version of the scale has been extensively tested and has been shown to yield reliable and valid responses [40, 41]. As described previously [39–41], we defined disability as needing personal assistance in performing one or more of the five essential activities (bathing, transferring, dressing, eating, and toileting) or being incontinent.

Our outcome measure is disability-free LE (DFLE), an easily interpretable metric for comparing population-level disability. Its calculation procedure is outlined below (see the “Statistical analyses” section). DFLE distinguishes between life-years spent free of disability and years with an ADL disability, providing a more nuanced view of population-level disability than simple estimates of LE or disability prevalence. It combines mortality and disability into a single measure, providing a convenient metric for measuring functional health at the population level [42].

### Sociodemographic variables

As described previously [16], several key sociodemographic variables including age, sex (men/women), residence (urban/rural), and educational attainment (1+ year/no formal schooling) were considered in this study. Urban/rural residence is strongly related to inequality in Chinese society with respect to healthcare services, income, economic growth, and infrastructural development and has been widely investigated in previous research [33, 43]. Educational attainment measures whether participants had completed formal schooling with two categories: 1+ year formal schooling and no any formal schooling. This distinction is appropriate for the particular Chinese cohorts under study, who have large percentages of illiterate individuals [32, 41]. To capture socioeconomic disparities [44], these two variables—residence and educational attainment—were further used for subgroup analyses in this study.

### Statistical analyses

Prior research on changes in population-level disability in China has relied on period comparisons—that is, measuring mortality and disability conditions in a population at different points in time and observing changes in the trends over time [19–23]. However, though this approach may be useful for monitoring aggregate trends in population-level disability, these results do not easily translate to the experience of any given birth cohort of individuals. In this study, we compared partial LE and DFLE across birth cohorts—that is, total LE, and LE in disability-free and disabled life, bounded between two ages. Additional file 1: Figure S1 displays a Lexis diagram showing the cohort comparison in ages 80–89 graphically. In brief (detailed descriptions are provided below), our approach estimated partial LE and DFLE using transition probabilities obtained from a 4- or 6-year snapshot of the life course of each 10-year birth cohort. We first estimated transition probabilities using longitudinal data from the observation period outlined in the dark dashed line (1998–2002 for the *earlier* cohort, 2008–2014 for the *later* cohort). These transition probabilities were then used to estimate partial LE and DFLE in oldest-old age ranges (80–89, 90–99, 100–105) for the birth cohorts. Finally, we compared partial LE and DFLE for the three paired age groups as mentioned above. Note that some birth cohorts are in the analysis more than once, as *earlier* cohorts in some analyses, and *later* cohorts in others. The CLHLS sample was periodically refreshed, and in practice, few individuals are present in more than one cohort comparison (less than 5% of the *earlier* cohort in the comparison of 80–89-year-olds were included in the *later* cohort in the comparison of 90–99-year-olds, and less than 2% of the *earlier* cohort in the comparison of 90–99-year-olds were included in the *later* cohort in the comparison of 100–105-year-olds).

More specifically, when generating the transition probabilities between nondisabled, disability, and death, our analysis method initially converted the CHLHS data to a person-year time scale, assuming that transitions between disability states occur at a random time between observations. We modeled these annual transition probabilities using a cumulative logistic regression model, stratified by initial disability state. The model includes age as a continuous predictor and sex, urban/rural residence, and educational attainment as binary variables, with the proportional odds assumption relaxed for sex, residence, and educational attainment [45]. We then generated matrices of age-specific transition probabilities for each combination of sex, residence, and educational attainment. Though (as discussed above) a small percentage of individuals are included in multiple age groups in our analyses, there were no individuals contributing person-years of observation to the *earlier* and *later* cohorts within each age group comparison. Models including an age^2^ and an age × sex interaction were also tested, but their coefficients were not significant at *α* = 0.05 and thus were removed in favor of the simpler model.

To generate estimates of partial LE and DFLE, we relied on microsimulation, a well-established tool in demographic research [46–50]. The transition probability matrices, estimated as described above, were applied via microsimulation to separate synthetic cohorts of 100,000 individuals in each cohort and age group (who have the same sex, residence, educational attainment, and initial disability state distribution as the observed cohorts). The resulting 100,000-person synthetic cohort was analyzed to estimate partial LE and DFLE. Point estimates shown were from transition probabilities estimated from the full sample. In the microsimulation approach, LE and DFLE estimates were not a deterministic function of the transition probabilities and instead resulted from a complex interplay between disability state, age, and individual characteristics as individuals move year-by-year through the simulation. Confidence intervals (CIs), which reflect both the uncertainty of the estimated parameters and the uncertainty from the microsimulation, were created by re-estimating the above analysis sequence using 499 bootstrap re-samples from each birth cohort under study. We took the central 95% of the distribution of these bootstrapped parameters as the 95% confidence interval and calculated non-parametric *P* values for differences in means between birth cohorts [51].

Inverse probability (IP) weights were included to correct for potential bias introduced from differential loss to follow-up. This method weighted complete cases (those not lost to follow-up) by the inverse of their probability of being a complete case and included the sociodemographic variables in Additional file 2: Table S1 and additional predictors that may influence the likelihood of loss to follow-up: province, marital status, ethnicity, co-residence with children, current smoking and alcohol use, self-reported hypertension, self-reported cardiovascular disease, and primary occupation (agricultural vs. non-agricultural) [52]. IP weights were generated separately by each age group and cohort included in these analyses. The weight generating models also included the cross-sectional survey sampling weight [53, 54]. All analyses were conducted in SAS 9.4 (SAS Institute, Cary, NC), and transition probability estimates were obtained using PROC SURVEYLOGISTIC, accounting for sample design and the IP weight.

## Results

Additional file 2: Table S1 presents the baseline characteristics of the two birth cohorts, including 7334 octogenarians, 7705 nonagenarians, and 5481 centenarians who were interviewed in 1998 or 2008. Across the three paired age groups, the *later* cohort had lower prevalence rates of disability than the *earlier* cohort. For example, 11.8% of octogenarians born 1919–1928 were disabled in 2008, while 17.3% of their age mates were disabled in 1998. We observed 4792 deaths in the *earlier* cohorts (1141 octogenarians, 1809 nonagenarians, and 1842 centenarians) during 4 years of follow-up and 7123 deaths in the *later* cohorts (1852 octogenarians, 3074 nonagenarians, and 2197 centenarians) over 6 years follow-up.

**Fig. 1:**
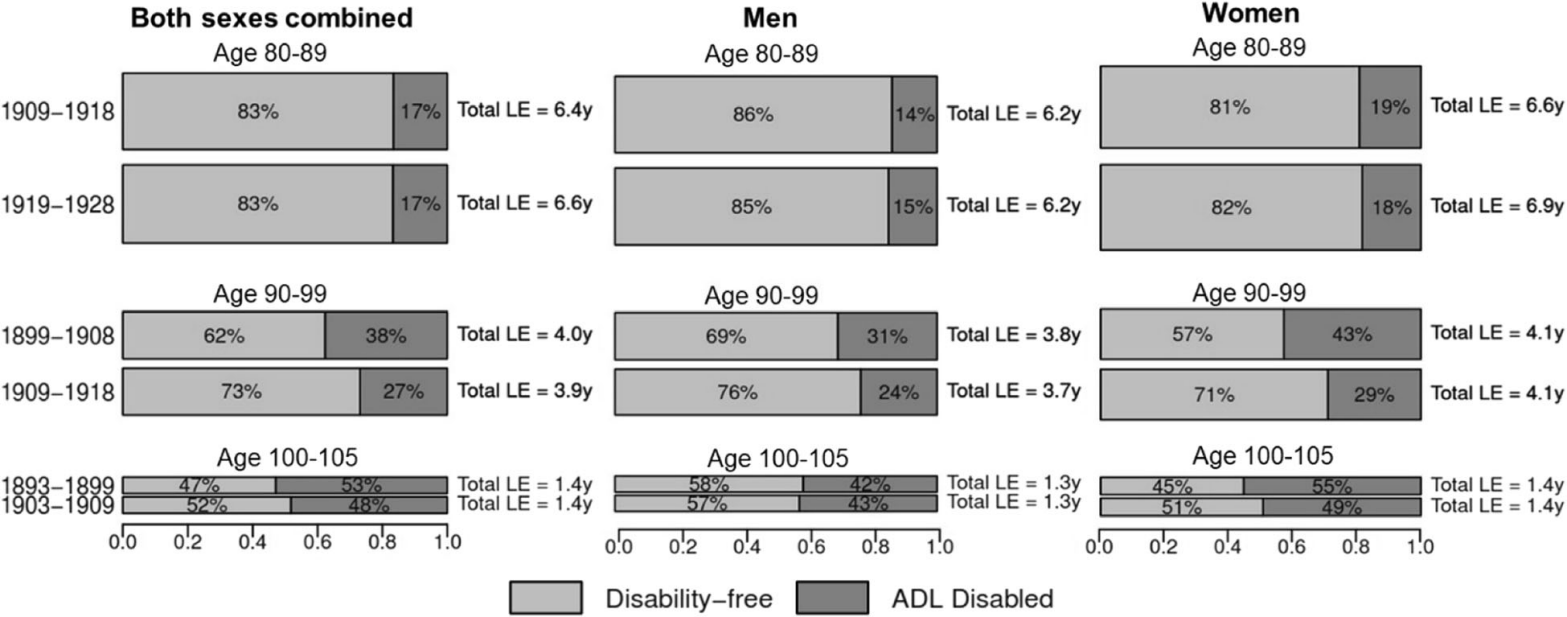
Proportion of partial life expectancy in ages 80–89, 90–99, and 100–105 spent disability-free and ADL disabled life by 10-year birth cohorts. LE, life expectancy; ADL, activity of daily living. The height and area of each bar is proportional to the partial LE in ages 80–89, 90–99, and 100–105, and the differently shaded areas represent the distribution of the LE across disability-free and ADL disabled life. The bars do not necessarily reflect the ordering of these life-years by disability states, as individuals in our analysis can recover and relapse between disability states, so not all years of disability are spent at the end of life

Table 1 and Fig. 1 present the results by comparing partial total, disability-free, and disabled LE in ages 80–89, 90–99, and 100–105 between successive 10-year birth cohorts, overall and by sex. More recently born octogenarians (born 1919–1928) had a longer partial LE between ages 80 and 89 than octogenarians born 1909–1918, with overall partial LE increasing by 0.20 years (*P* = 0.044). Most of this gain occurred among women, who experienced an increase in partial LE of 0.32 years (*P* = 0.012) across the two birth cohorts. This increase in LE was entirely comprised of a 0.32-year rise in partial DFLE (*P* = 0.004). In Fig. 1, we observed that, though partial LE in ages 80–89 increased between these two birth cohorts, there was little evidence of a compression of morbidity—that is, the proportion of life spent disability-free in these ages was little changed. Although no increases in partial LE were observed among nonagenarians or centenarians, partial DFLE increased in these persons across successive 10-year birth cohorts, with a gain of 0.41 years (*P* < 0.001) among nonagenarians and 0.07 years (*P* = 0.050) among centenarians. As shown in Fig. 1, there was strong evidence that a compression of morbidity happened over successive cohorts among nonagenarians and centenarians—more recently born cohorts are living substantially more of these years disability-free. Women appeared to be experiencing a more rapid compression of disability across cohorts than men—partial DFLE rose by 0.58 years (*P* < 0.001) among nonagenarian women and by 0.09 years (*P* = 0.016) among centenarian women, compared with smaller gains of 0.18 disability-free years (*P* = 0.054) for nonagenarian men and negligible change for centenarian men.

**Table 1:** Partial total, disability-free, and disabled life expectancy in ages 80–89, 90–99, and 100–105 across 10-year birth cohorts, overall and by sex

	Octogenarians (aged 80–89)		Diff	*P* value
	Birth cohort			
	1909–1918	1919–1928		
Overall				
Total	6.38 [6.1–6.59]	6.58 [6.36–6.82]	0.20	0.044
Disability-free	5.32 [5.01–5.5]	5.47 [5.2–5.72]	0.15	0.110
ADL disabled	1.06 [0.95–1.24]	1.1 [0.96–1.28]	0.04	0.740
Men				
Total	6.14 [5.8–6.42]	6.22 [5.93–6.49]	0.08	0.566
Disability-free	5.29 [4.91–5.55]	5.28 [4.93–5.55]	− 0.01	0.950
ADL disabled	0.86 [0.73–1.03]	0.94 [0.82–1.16]	0.08	0.226
Women				
Total	6.62 [6.27–6.9]	6.94 [6.67–7.26]	0.32	0.012
Disability-free	5.35 [4.98–5.59]	5.67 [5.38–6.02]	0.32	0.004
ADL disabled	1.27 [1.1–1.49]	1.27 [1.04–1.47]	0.00	0.592
	Nonagenarians (aged 90–99)		Diff	*P* value
	Birth cohort			
	1899–1908	1909–1918		
Overall				
Total	3.96 [3.78–4.13]	3.94 [3.81–4.1]	− 0.02	0.932
Disability-free	2.47 [2.26–2.69]	2.88 [2.75–3.06]	0.41	< 0.001
ADL disabled	1.49 [1.34–1.64]	1.06 [0.95–1.17]	− 0.43	< 0.001
Men				
Total	3.83 [3.6–4.08]	3.71 [3.55–3.9]	− 0.12	0.310
Disability-free	2.65 [2.42–2.9]	2.83 [2.71–3.05]	0.18	0.054
ADL disabled	1.18 [1.03–1.32]	0.88 [0.76–0.99]	− 0.30	< 0.001
Women				
Total	4.07 [3.84–4.27]	4.1 [3.95–4.29]	0.03	0.606
Disability-free	2.33 [2.11–2.55]	2.91 [2.75–3.1]	0.58	< 0.001
ADL disabled	1.74 [1.55–1.93]	1.19 [1.06–1.33]	− 0.55	< 0.001
	Centenarians (aged 100–105)		Diff	*P* value
	Birth cohort			
	1893–1899	1903–1909		
Overall				
Total	1.38 [1.31–1.47]	1.4 [1.31–1.48]	0.02	0.756
Disability-free	0.65 [0.59–0.72]	0.72 [0.66–0.82]	0.07	0.050
ADL disabled	0.73 [0.67–0.79]	0.67 [0.59–0.75]	− 0.06	0.088
Men				
Total	1.3 [1.16–1.44]	1.32 [1.18–1.47]	0.02	0.744
Disability-free	0.76 [0.63–0.88]	0.75 [0.64–0.91]	− 0.01	0.832
ADL disabled	0.54 [0.46–0.66]	0.56 [0.46–0.66]	0.02	0.872
Women				
Total	1.41 [1.32–1.49]	1.42 [1.32–1.51]	0.01	0.794
Disability-free	0.63 [0.57–0.7]	0.72 [0.65–0.82]	0.09	0.016
ADL disabled	0.78 [0.71–0.85]	0.7 [0.62–0.78]	− 0.08	0.052

Data are life expectancy in years unless specified, with the 95% confidence interval in brackets after point estimate. Models adjusted for age, sex (only for overall), education, and urban/rural residence. *Abbreviations*: *Diff* difference, *ADL* activity of daily living

**Fig. 2:**
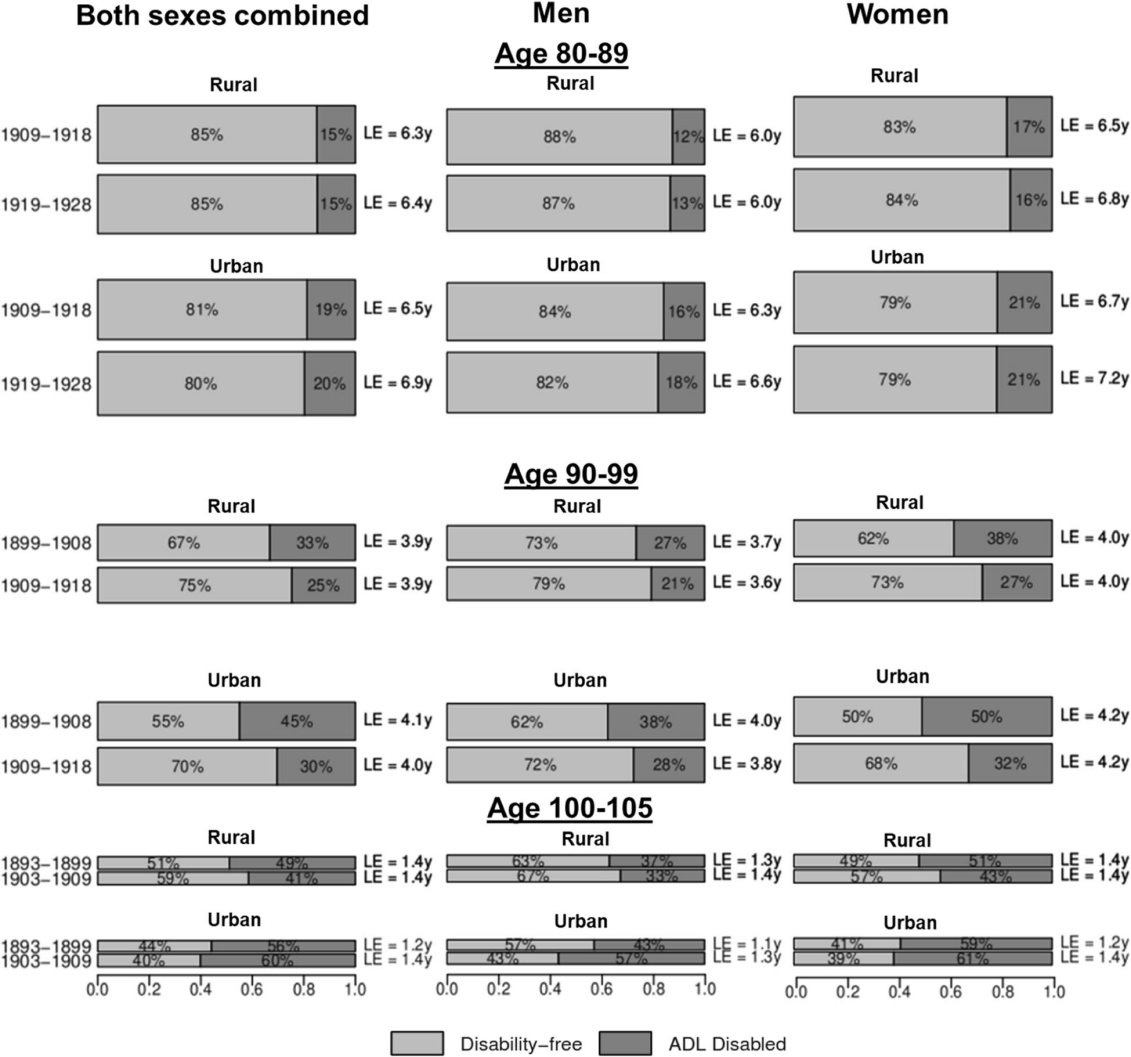
Proportion of partial life expectancy in ages 80–89, 90–99, and 100–105 spent disability-free and ADL disabled life by 10-year birth cohorts, by urban/rural residence. LE, life expectancy; ADL, activity of daily living. The height and area of each bar is proportional to the partial overall LE in ages 80–89, 90–99, and 100–105, and the differently shaded areas represent the distribution of the LE across disability-free and ADL disabled life. The bars do not necessarily reflect the ordering of these life-years by disability states, as individuals in our analysis can recover and relapse between disability states, so not all years of disability are spent at the end of life

**Fig. 3:**
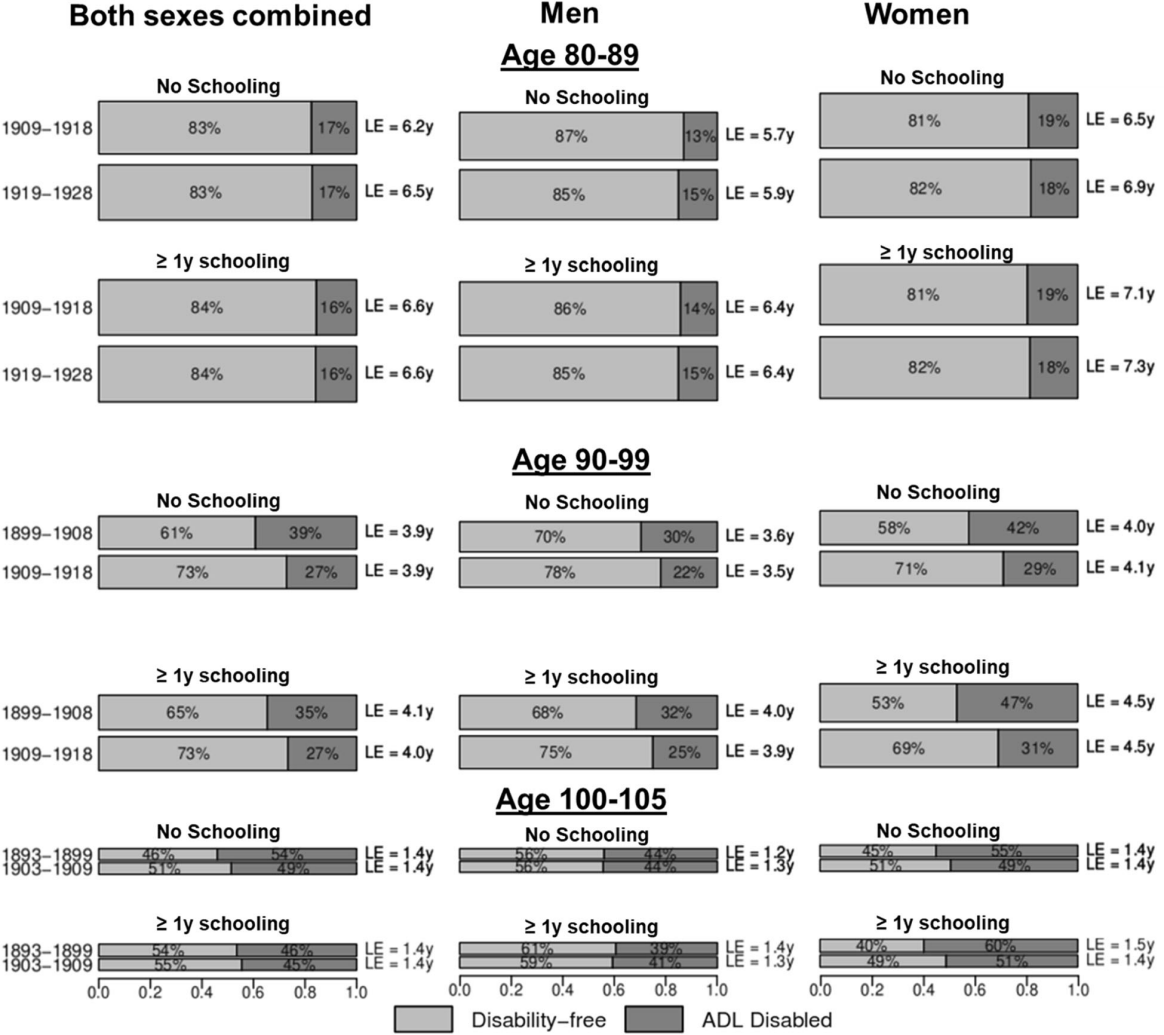
Proportion of partial life expectancy in ages 80–89, 90–99, and 100–105 spent disability-free and ADL disabled life by 10-year birth cohorts, by formal schooling attainment. LE, life expectancy; ADL, activity of daily living. The height and area of each bar is proportional to the partial overall LE in ages 80–89, 90–99, and 100–105, and the differently shaded areas represent the distribution of the LE across disability-free and ADL disabled life. The bars do not necessarily reflect the ordering of these life-years by disability states, as individuals in our analysis can recover and relapse between disability states, so not all years of disability are spent at the end of life

Partial LE estimates and the proportion of life spent disability-free and disabled by urban/rural residence and education are presented in Figs. 2 and 3, respectively, with detailed results in Additional file 3: Table S2, Additional file 4: Table S3, Additional file 5: Table S4, Additional file 6: Table S5, Additional file 7: Table S6, and Additional file 8: Table S7. Urban residents aged 80–89 lived 0.39 years longer (*P* < 0.001) in the *later* cohort compared to the *earlier* cohort, with the majority of these additional life-years spent disability-free (0.25 years, *P* = 0.030). In contrast, rural 80–89-year-old individuals saw a much smaller increase in partial LE (0.10 years, *P* = 0.392) and DFLE (0.10 years, *P* = 0.428). Though partial LE increased faster for urban residents, the proportions of remaining life spent disability-free and disabled were little changed between the two cohorts. No urban/rural differences in partial LE were seen between birth cohorts in the 90–99 or 100–105 age ranges. Both urban and rural residents in ages 90–99 experienced gains in partial DFLE across successive birth cohorts, though this gain was larger among urban residents (0.54 years, *P* < 0.001) than in rural residents (0.33 years, *P* < 0.001). As seen in Fig. 2, the urban population gained proportionately more years of disability-free life in ages 90–99, with the proportion of life-years lived disability-free rising from 55 to 70% across successive cohorts. In contrast, partial DFLE increased by 0.12 years (*P* < 0.001) among rural 100–105-year-old individuals between the *earlier* and *later* cohorts, compared to 0.02 years (*P* = 0.602) among urban residents. An urban/rural disparity in partial LE was evident in octogenarians and nonagenarians, where partial LE for rural residents in the *later* cohort trailed behind total LE for urban residents born 10 years prior.

Partial LE and DFLE gains across cohorts in ages 80–89 were largest in the population with no formal schooling (Fig. 3)—partial LE increased by 0.31 years (*P* = 0.006) between the two birth cohorts for those without formal schooling. Though gains in partial LE were stronger among those with no education in ages 80–89, LE in this group still trailed behind those who received some formal schooling, particularly among men. Figure 3 suggests that there was little compression of disability happening in either schooling group in ages 80–89. Partial LE was little changed across cohorts in ages 90–99 and 100–105. Those with no formal schooling saw a larger rise in partial DFLE of 0.47 years (*P* < 0.001) in ages 90–99 (compared to 0.27 years [*P* = 0.008] for those with some formal schooling) and 0.09 years (*P* = 0.028) in ages 100–105 (compared to no change for those with some formal schooling). For both those with and without schooling, evidence for the compression of disability was stronger in women than in men across successive cohorts.

## Discussion

Understanding trends in disability and longevity among the oldest-old is of great importance for policy and planning purposes in aging countries worldwide. To the best of our knowledge, this is the first comparative and longitudinal modeling analysis to evaluate recent trends in population-level disability among the Chinese oldest-old taking the cohort dynamics of disability into consideration. By comparing partial LE and DFLE across successive 10-year birth cohorts in ages 80–89, 90–99, and 100–105, we demonstrated that more recently born cohorts are living more disability-free years in oldest-old ages. Women and less educated individuals are experiencing a faster compression of disability, but the urban-rural disparity in disability is widening among China’s oldest-old.

### Comparisons with previous studies in China and other countries

Our longitudinal findings are largely consistent with a previous study that compared birth cohorts cross-sectionally in China [16], though some differences are noted. Comparing cohorts between only the 1998 and 2008 waves, Zeng et al. also found a decrease in the prevalence of ADL disability, which aligns with our finding of increases in partial DFLE across birth cohorts. Zeng et al. found a slight decline in mortality across birth cohorts at all ages, while we only found appreciable increases in partial LE in ages 80–89. However, Zeng’s study used a modeling approach (parametric survival models) that prioritized precision in measuring the hazard of mortality, a substantially different approach than the generalized logistic hazard model used in our analyses to measure transitions between multiple outcome states. Though our models found no significant differences in partial LE between cohorts at ages 90–99 and 100–105, the CIs around these estimates are fairly wide. As such, our results do not contradict those of Zeng et al., but rather seek to answer a different question. Future MSLT work could potentially utilize recent advances in parametric multistate survival models to improve model fit by specifying different underlying hazards for different state transitions [55], but more dedicated methodological work is needed to adapt these methods to demographic applications. In addition, our finding on the more rapid improvements in LE and DFLE among the urban resident population indirectly supports Zeng’s argument that the changes of ADL are mostly based on contextual changes due to the rapid economic development in urban areas [16]. A more detailed discussion of potential factors leading to the observed increases in LE and DFLE in China’s oldest-old is discussed below.

Although direct comparisons of DFLE across different countries are somewhat problematic due to methodological differences (e.g., MSLTs vs Sullivan’s method) and differences in outcome measures of disability and/or morbidity [56], it is useful to place our findings in the context of other research on DFLE in the oldest-old. Findings from Great Britain [57], Denmark [58], and the USA [59] suggest that oldest-old individuals in these countries are living longer and with fewer years of disability, than in previous years, though there is some evidence of countervailing trends in younger ages [60]. However, trends in the oldest-old have been mixed in other countries; for example, in Sweden, one study found that oldest-old individuals are living longer and with less disability [61], while another found stagnation in ADL disability and declines in physical functioning [62]. Research in both Japan [63] and Singapore [64] has found increasing LE over time among those aged 85+, but declining DFLE as a portion of the remaining life.

### Key factors impacting LE and DFLE

Prior research suggests several key factors impacting LE and DFLE at oldest-old ages: differing levels of mortality selection to oldest-old ages [65]; differences in life-course stressors, diseases, and health behaviors [66, 67]; and access to and quality of healthcare services at oldest-old ages [68]. In this study, we aimed to describe cohort differences, but were not able to disentangle the various roles of these key factors. As reliable mortality statistics in China have only become available in recent years, ascertaining changes in the level of mortality selection of the older Chinese cohorts in our study is difficult. In the sections below, we discussed other contextual trends in Chinese society that may have contributed to our findings.

The Chinese oldest-old have witnessed great socioeconomic and epidemiological transitions throughout their life course. These cohorts underwent the Sino-Japanese and domestic wars (1937–1949) in childhood, experienced famine and social disruption in the Great Leap Forward (1958–1961) and the Cultural Revolution (1966–1976), and lived through a massive shift from a centrally planned to a market-based economy in their adulthood [20]. This shift brought increasing inequality and disruption of healthcare systems across the country, leading to less access to care in the late 1990s, particularly in rural regions [20, 69, 70]. Nevertheless, since the epidemic of severe acute respiratory syndrome in 2003, the implementation of the New Rural Cooperative Medical System and the Urban Medical Schemes, coupled with policies and benefits targeting the oldest-old since 1995, has made medical care more accessible and affordable, though the reimbursement level is still low [71, 72].

In recent years, policymakers in China have dedicated significant resources to improving health and well-being of older adults. A central tenant of these recent healthcare reforms was to promote equity [73], and our results suggest that these efforts have begun to show successes. Women and those without formal schooling, often thought of as disadvantaged subgroups in China, were more likely to experience a compression of disability in this study, consistent with results from period-based comparisons [23, 31]. The disabling effects of some major diseases (e.g., stroke and cardiometabolic diseases) have also declined during the last few decades in China [21, 74, 75]. The empirical observation of gains in DFLE in this study may partially reflect the success of these investments/programs for improving functional health in the oldest-old.

Differences in access to healthcare may also be one of the explanations for the increasing urban-rural disparity in disability [33, 43]. Though the current policy environment has explicitly prioritized rural healthcare development, this is a very recent development. Over the life course of these oldest-old individuals, substantial disparities existed in access to and quality of healthcare between urban and rural residents. When the collectively funded welfare programs (Cooperative Medical Scheme, or *hezuo yiliao*) were abandoned in most rural areas in the early 1980s, healthcare became predominantly employment-based. Medical insurance coverage varied considerably by residence (rural, 7.4%; urban, 36.4%) [76]. This gap in coverage remained wide by 2003, when the New Rural Cooperative Medical System was launched. Care provided through this scheme is far from comprehensive, however [77]. Older adults in rural areas have less access to preventative care and/or timely treatment and are more likely to cite financial constraints as a barrier to accessing care [33]. Conversely, urban residents have benefited more from recent programs increasing access to medical care, including the Urban Employer-sponsored Medical Scheme and the Urban Resident Medical Scheme.

The broader economic and social trends may influence the changes of LE and DFLE, e.g., disadvantaging rural oldest-old individuals. A substantial urban-rural gap in economic development has existed for decades in China and has even widened in recent years with rapid socioeconomic development in urban areas [78]. This growing gap may directly explain a substantial portion of the urban/rural inequality found in our study. In addition, the high rates of rural to urban migration of younger adults, largely for increased economic opportunities, may reduce available care and support networks of oldest-old adults. Previous research has found that these “left behind” older adults (where an adult child has migrated away to an urban area) are more likely to experience poor health and that this effect was amplified for those in low-income households and for older individuals [79]. Finally, changes in health behaviors may contribute to our findings. For instance, the prevalence of smoking in Chinese older men has been decreasing in recent decades [80].

Though the above contextual information on China’s oldest-old suggests that these changing circumstances—increasing healthcare access, improvements in economic well-being, and improving health behaviors in earlier life—have led to overall improvements in functioning among successive generations of the oldest-old, our current analyses are not able to disentangle these interrelated sources of variation. Changes in LE and DFLE are the end result of a series of complex processes, and we believe that our results are unlikely to be driven by any one of these contextual changes in isolation. Nevertheless, we do think that our findings are helpful in identifying policy strategies that other countries could follow in supporting the health and well-being of their oldest-old populations. Our findings suggest that the concerted efforts of China’s policymakers in improving healthcare accessibility, though not without flaws, have contributed to improvements in well-being at these ages. However, the growing urban-rural divide suggests that policymakers seeking to learn from China’s successes be cognizant of the need to ensure equality of access to care improvements across population subgroups.

### Strengths and limitations

One of the main strengths of our analysis is the focus on understanding changes in disability across birth cohorts. With a few notable exceptions [60, 81–83], most work investigating compression of disability has relied on period-based comparisons which aggregate information across a large number of birth cohorts. Though this approach may be useful for monitoring aggregate trends in population-level disability, these results do not easily translate to the experience of any given cohort of individuals. Measuring changes in disability in cohort perspective is preferable, providing results that match more closely with the lived experience of individuals in a population [82, 83]. Indeed, previous work has found that period estimates of DFLE have relatively poor correspondence with cohort DFLE [83]. Our analyses, centered on evaluating how partial LE and DFLE are changing within age groups over birth cohorts, provide information that is more directly applicable to understanding trends in population health over successive generations. Another strength is that our analyses use the largest nationally representative cohort of the oldest-old in the world, providing us with a unique opportunity to examine disability changes in a substantial fraction of the global oldest-old.

This study also has several limitations. First, our analyses follow a first-order Markov chain and are thus not state-duration-dependent—that is, transition probabilities are not adjusted by duration of stay in a given state. Individuals who experience a disability transition between waves of data collection are assumed to experience only a single transition during the period between surveys, which likely misses shorter-term transitions between disability statuses. The Markov assumption that no unobserved transitions occur before death is a particularly problematic one, as this makes the somewhat unrealistic assumption that individuals who were observed as disability-free and die before the next wave experienced no disability prior to death [84]. To explore the potential for this assumption to bias our results, we conducted a sensitivity analysis wherein we re-allocated a portion of the life-year prior to death for initially disability-free individuals who died before the next wave, with the proportion of this year allocated as disabled generated by a random draw from the uniform distribution. As expected, LE spent disabled increases in the adjusted estimates, and LE spent disability-free declines (Additional file 9: Table S8). Differences are larger for men than for women, largely as a function of fewer men reporting an ADL disability in later life. Nonetheless, the substantive findings on cohort differences in the adjusted results follow closely to those from the standard, unadjusted MSLT model.

Second, the time between interviews varied between the two periods under study, with a 2-year period between the 1998, 2000, and 2002 waves of CLHLS data collection and a 3-year period elapsing between the 2008, 2011, and 2014 waves of data collection. This difference in time periods may lead us to miss proportionately more short-term transitions in the 2008–2014 period, lowering the predicted transition rates of both onset of disability and recovery from disability. Based on prior empirical and simulation studies, these downward biases are in large part offsetting [85], and the 1-year difference in observation periods should still produce unbiased partial total LE and DFLE estimates [86].

Third, as mentioned previously, our modeling approach prioritizes estimating DFLE as the primary outcome, which leads to less precision in our estimation of partial total LE compared to other modeling approaches (such as standard hazard modeling). Fourth, the available CLHLS analysis weights are cross-sectional in nature and thus inapplicable to our birth cohort-based analyses. Without cohort sampling weights, our analyses must thus be understood as representing the CLHLS study cohort, which may not precisely match the experience of the Chinese oldest-old population as a whole. Fifth, due to the sample size, we were not able to perform more subgroup analyses for socioeconomic inequality except residence and education. Finally, the measure of disability is limited to ADL disability, excluding instrumental ADL (IADL) disability and/or functional limitation. Given the amount of overlap between ADL and IADL disability in the oldest-old, expanding our outcome measure to include IADL disability would be unlikely to substantially alter our findings. However, we suggest caution when generalizing our results to other facets of health, as trends in specific morbidities or lower-level functional limitations may not exactly follow the patterns of ADL disability. Our reliance on a single measure of disability did not permit us to evaluate whether disability in China is following the dynamic equilibrium scenario, in which severe (ADL) disability is declining, but less severe disability is increasing [16, 17].

## Conclusions

These findings show that China’s oldest-old are living with less disability as a whole over successive generations. LE is increasing among octogenarians, and a compression of disability is occurring among nonagenarians and centenarians. However, we also found a widening gap in partial LE and DFLE between rural and urban areas. China may need to develop more specific policies to alleviate health inequality throughout the population, particularly in improving access to healthcare and supports in rural communities.

## Additional files

Additional file 1: **Figure S1.** Lexis diagram showing cohort comparison for octogenarians (aged 80–89). (DOCX 287 kb)

Additional file 2: **Table S1.** Baseline characteristics of the birth cohorts, overall and by sex. (DOCX 15 kb)

Additional file 3: **Table S2.** Partial total, disability-free, and disabled life expectancy in ages 80–89, 90–99, and 100–105 across 10 years birth cohorts, both sexes combined, by urban/rural residence and schooling. (DOCX 18 kb)

Additional file 4: **Table S3.** Partial total, disability-free, and disabled life expectancy in ages 80–89, 90–99, and 100–105 across 10 years birth cohorts in men, by urban/rural residence and schooling. (DOCX 18 kb)

Additional file 5: **Table S4.** Partial total, disability-free, and disabled life expectancy in ages 80–89, 90–99, and 100–105 across 10 years birth cohorts in women, by urban/rural residence and schooling. (DOCX 18 kb)

Additional file 6: **Table S5.** Proportion of remaining partial life expectancy spent disability-free and disabled in ages 80–89, 90–99, and 100–105 across 10 years birth cohorts, both sexes combined. (DOCX 16 kb)

Additional file 7: **Table S6.** Proportion of remaining partial life expectancy spent disability-free and disabled in ages 80–89, 90–99, and 100–105 across 10 years birth cohorts, men. (DOCX 16 kb)

Additional file 8: **Table S7.** Proportion of remaining partial life expectancy spent disability-free and disabled in ages 80–89, 90–99, and 100–105 across 10 years birth cohorts, women. (DOCX 16 kb)

Additional file 9: **Table S8.** Comparison between standard MSLT estimates (Unadjusted) and estimates imputing a period of disability at end-of-life (Adjusted), ages 80–89, 90–99, and 100–105 across 10 years birth cohorts (DOCX 19 kb)

## Abbreviations

ADL: Activities of daily living
CI: Confidence interval
CLHLS: Chinese Longitudinal Healthy Longevity Survey
DFLE: Disability-free life expectancy
IADL: Instrumental activities of daily living
IP: Inverse probability
LE: Life expectancy
MSLT: Multistate life table
