# Supplementary figures and images for: Are China’s oldest-old living longer with less disability? A longitudinal modeling analysis of birth cohorts born 10 years apart
Source: BMC Med. 2019 Feb 1;17:23. doi: 10.1186/s12916-019-1259-z (PMC6357399; doi:10.1186/s12916-019-1259-z)

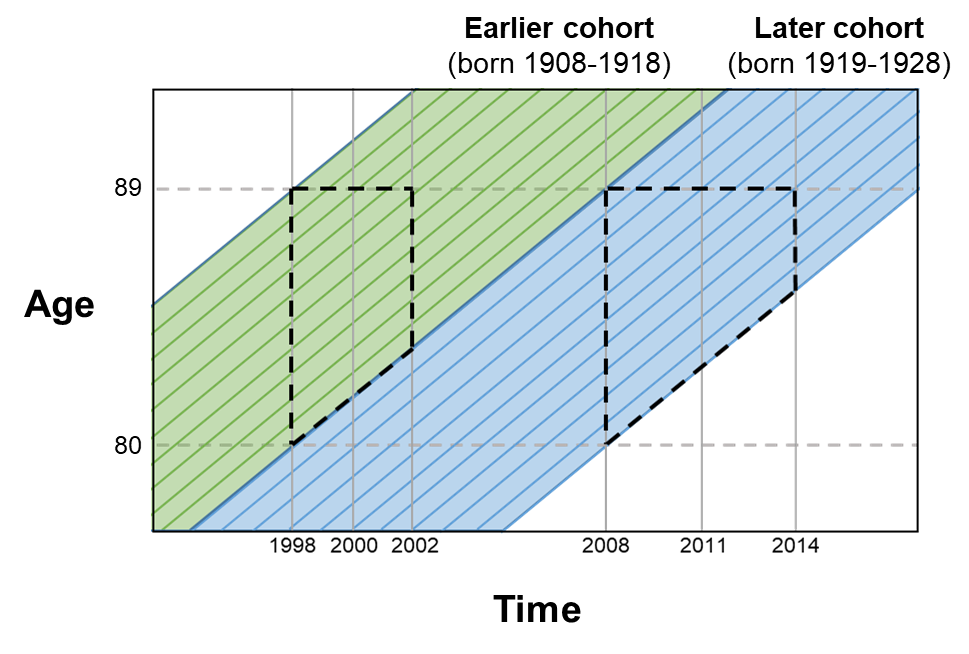


**Figure S1.** Lexis diagram showing cohort comparison for octogenarians (aged 80-89).

Supplement: Supplementary file 1 — Figure S1. Lexis diagram showing cohort comparison for octogenarians (aged 80–89). (DOCX 287 kb) [file 12916_2019_1259_MOESM1_ESM.docx]
