# Supplementary material for: Are China’s oldest-old living longer with less disability? A longitudinal modeling analysis of birth cohorts born 10 years apart
Source: BMC Med. 2019 Feb 1;17:23. doi: 10.1186/s12916-019-1259-z (PMC6357399; doi:10.1186/s12916-019-1259-z)
Supplement: Supplementary file 2 — Table S1. Baseline characteristics of the birth cohorts, overall and by sex. (DOCX 15 kb) [file 12916_2019_1259_MOESM2_ESM.docx]

**Table S1.** Baseline characteristics of the birth cohorts, overall and by sex

|  | **Octogenarians (Aged 80-89)** | |  | **Nonagenarians (Aged 90-99)** | |  | **Centenarians (Aged 100-105)** | |
| --- | --- | --- | --- | --- | --- | --- | --- | --- |
| Baseline interview year | 1998 | 2008 |  | 1998 | 2008 |  | 1998 | 2008 |
| Birth cohort | 1909-1918 | 1919-1928 |  | 1899-1908 | 1909-1918 |  | 1893-1899 | 1903-1909 |
| Sample size | 3,242 | 4,092 |  | 2,993 | 4,712 |  | 2,406 | 3,075 |
| **Age, Mean (standard deviation)** | |  |  |  |  |  |  |  |
| Both sexes combined | 84.0 (2.7) | 84.5 (2.9) |  | 93.6 (2.9) | 93.2 (2.7) |  | 101.0 (1.4) | 101.6 (1.6) |
| Men | 84.0 (2.8) | 84.5 (2.9) |  | 93.4 (2.9) | 93.0 (2.6) |  | 100.8 (1.4) | 101.5 (1.5) |
| Women | 84.0 (2.7) | 84.6 (2.9) |  | 93.8 (3.0) | 93.4 (2.8) |  | 101.0 (1.4) | 101.7 (1.6) |
| **Residence, rural, n (%)** | |  |  |  |  |  |  |  |
| Both sexes combined | 1,823 (56.2) | 2,522 (61.6) |  | 1,892 (63.2) | 2,765 (58.7) |  | 1,723 (71.6) | 1,901 (61.8) |
| Men | 933 (57.4) | 1,287 (61.9) |  | 831 (63.3) | 1,108 (56.8) |  | 352 (69.7) | 346 (53.7) |
| Women | 890 (55.0) | 1,235 (61.3) |  | 1,061 (63.1) | 1,657 (60.0) |  | 1,371 (72.1) | 1,555 (64.0) |
| **≥1 years of educational attainment*, n (%)** | |  |  |  |  |  |  |  |
| Both sexes combined | 1,428 (44.2) | 1,584 (38.8) |  | 968 (32.5) | 1,411 (30.1) |  | 375 (15.7) | 444 (14.5) |
| Men | 1,096 (67.5) | 1,245 (60.1) |  | 783 (59.8) | 1,065 (54.8) |  | 252 (50.2) | 273 (42.8) |
| Women | 332 (20.6) | 339 (16.9) |  | 185 (11.1) | 346 (12.6) |  | 123 (6.5) | 171 (7.1) |
| **Disability†, n (%)** |  |  |  |  |  |  |  |  |
| Both sexes combined | 557 (17.3) | 481 (11.8) |  | 1,116 (37.4) | 1,270 (27.0) |  | 1,491 (62.2) | 1,599 (52.0) |
| Men | 244 (15.1) | 224 (10.8) |  | 398 (30.5) | 454 (23.3) |  | 277 (55.0) | 312 (48.5) |
| Women | 313 (19.4) | 257 (12.8) |  | 718 (42.9) | 816 (29.6) |  | 1,214 (64.1) | 1,287 (52.9) |
| **Individual ADL in both sex combined**^‡^**, n (%)** | |  |  |  |  |  |  |  |
| Bathing | 446 (13.8) | 423 (10.3) |  | 964 (32.2) | 1,160 (24.6) |  | 1,307 (54.4) | 1,495 (48.6) |
| Dressing | 182 (5.6) | 210 (5.1) |  | 492 (16.4) | 630 (13.4) |  | 829 (34.5) | 818 (26.6) |
| Toileting | 208 (6.4) | 216 (5.3) |  | 544 (18.2) | 649 (13.8) |  | 913 (38.0) | 848 (27.6) |
| Transferring | 161 (5.0) | 180 (4.4) |  | 457 (15.3) | 570 (12.1) |  | 796 (33.1) | 735 (23.9) |
| Continence | 132 (4.1) | 90 (2.2) |  | 254 (8.5) | 307 (6.5) |  | 366 (15.2) | 406 (13.2) |
| Eating | 130 (3.2) | 146 (3.6) |  | 341 (11.4) | 415 (8.8) |  | 574 (23.9) | 522 (17.0) |

ADL, activities of daily living. Values are numbers (percentages) unless stated otherwise.

^*^ The number of missing data on educational attainment was 44 for both *earlier* and *later* cohorts (i.e., 80-105 years in 1998 and 2008).

^†^ The number of missing data on disability was 34 in *earlier* cohort and 1 in the *later* cohort.

^‡^The number of missing data was 11 for bathing, 3 for transferring, 5 for dressing, 22 for eating, 4 for toileting, and 7 for continence in the *earlier* cohort. The number of missing data was 1 for bathing, and 0 for other five activities in the *later* cohort.
