# Supplementary material for: Are China’s oldest-old living longer with less disability? A longitudinal modeling analysis of birth cohorts born 10 years apart
Source: BMC Med. 2019 Feb 1;17:23. doi: 10.1186/s12916-019-1259-z (PMC6357399; doi:10.1186/s12916-019-1259-z)
Supplement: Supplementary file 5 — Table S4. Partial total, disability-free, and disabled life expectancy in ages 80–89, 90–99, and 100–105 across 10 years birth cohorts in women, by urban/rural residence and schooling. (DOCX 18 kb) [file 12916_2019_1259_MOESM5_ESM.docx]

**Table S4:** Total, active, and disabled life expectancy in ages 80-89, 90-99, and 100-105 across 10 years birth cohorts in women, by urban/rural residence and schooling

Diff, difference; ADL, activities of daily living. Data are life expectancy in years unless specified, with the 95% confidence interval in brackets after point estimate.
