# Supplementary material for: Are China’s oldest-old living longer with less disability? A longitudinal modeling analysis of birth cohorts born 10 years apart
Source: BMC Med. 2019 Feb 1;17:23. doi: 10.1186/s12916-019-1259-z (PMC6357399; doi:10.1186/s12916-019-1259-z)
Supplement: Supplementary file 7 — Table S6. Proportion of remaining partial life expectancy spent disability-free and disabled in ages 80–89, 90–99, and 100–105 across 10 years birth cohorts, men. (DOCX 16 kb) [file 12916_2019_1259_MOESM7_ESM.docx]

**Table S6.** Proportion of remaining life expectancy spent disability-free and disabled in ages 80-89, 90-99, and 100-105 across 10 years birth cohorts, by urban/rural residence and schooling, men

Diff, difference; ADL, activities of daily living. Data are proportions unless specified, with the 95% confidence interval in brackets after point estimate.
