# Supplementary material for: Are China’s oldest-old living longer with less disability? A longitudinal modeling analysis of birth cohorts born 10 years apart
Source: BMC Med. 2019 Feb 1;17:23. doi: 10.1186/s12916-019-1259-z (PMC6357399; doi:10.1186/s12916-019-1259-z)
Supplement: Supplementary file 9 — Table S8. Comparison between standard MSLT estimates (Unadjusted) and estimates imputing a period of disability at end-of-life (Adjusted), ages 80–89, 90–99, and 100–105 across 10 years birth cohorts (DOCX 19 kb) [file 12916_2019_1259_MOESM9_ESM.docx]

**Table S8.** Comparison between standard MSLT estimates (Unadjusted) and estimates imputing a period of disability at end-of-life (Adjusted), ages 80-89, 90-99, and 100-105 across 10 years birth cohorts

Diff, difference; ADL, activities of daily living; MSLT, multistate life table. Data are life expectancies in years unless specified. Unadjusted column represents estimates generated with the standard Markov assumption of no unobserved state transitions. Adjusted column represents estimates generated from a model that imputes a period of ADL disability prior to death, with length of disability randomly drawn from the uniform distribution.
